# Correlation Analysis between Higher Education Level and College Students' Public Mental Health Driven by AI

**DOI:** 10.1155/2022/4204500

**Published:** 2022-09-12

**Authors:** Yinying Cai, Ling Tang

**Affiliations:** Chongqing University of Education, Chongqing 400067, China

## Abstract

Generally, there is a certain correlation between the level of higher education and the public mental health of college students. Traditionally, questionnaires and literature research methods are used to analyze the correlation between mental health and higher education, but these methods are always limited by many factors, such as resource conditions, survey paths, theoretical framework, and technical means. In recent years, with the rapid development and application of artificial intelligence technology, a new direction of analyzing the correlation between higher education level and college students' public mental health has been given. The artificial intelligence method makes the correlation analysis change from subjective to big data algorithm evaluation, which can make up for the shortcomings and inefficiency of traditional methods, truly analyze the degree of correlation, and put forward exact solutions, which is of great significance for further evaluating and monitoring the public mental health of college students in higher education. This study first analyzes different AI algorithms and determines to use convolution neural network and random forest algorithm to establish an AI correlation model. After testing and data analysis, the established model has an accuracy of 87.5% in the determination and analysis of correlation. Compared with support vector machine (SVM) and backpropagation (BP) neural network algorithm, it has a higher recognition accuracy.

## 1. Introduction

In recent years, with the increasing pressure and challenges faced by college students in academic, employment and life, students with weak psychological pressure tolerance are vulnerable to the influence of external environment and events, accumulate excessive negative emotions, and produce negative and sad emotions. If they cannot find the right way to vent their emotions, over time, students may fall into serious psychological problems [1]. However, many colleges and universities pay more attention to the higher education of college students, and the solution to the mental health problems of college students is very weak. Many colleges and universities pay more attention to the higher education of college students, but pay less attention to the mental health problems of college students, and their coping styles are very weak. Therefore, the level of higher education and the public mental health of college students are two important factors that affect the growth and development of college students. No matter which aspect has problems, it will have a certain negative impact on the other side and the personal development of college students [2]. It is conceivable that at the university stage, students' basic abilities, and psychological endurance have a certain foundation. With it, it is necessary to solve the public mental health problems of college students from a professional perspective, because there is always a certain correlation between higher education and mental health. Analyzing the correlation between the two plays an important role in the formation of college students' values and correct ideological cognition.

In the research on the correlation between the level of higher education and the public mental health of college students, Ba [2] believes that the mental health of college students is the basis for carrying out higher education, and higher education helps to maintain the mental state of college students and guide and ensure the effectiveness of mental health education. It also believes that there are some problems in the implementation of higher education, such as the lack of effective teaching organization planning, the relatively simple teaching organization planning, and the lack of comprehensive solutions to the problems of college students. Finally, it is suggested that higher education should do a good job in organization and planning, flexibly use diverse teaching organization methods, and pay attention to teaching practice to solve the actual psychological problems of college students. Wu [3] tested the mental health status of 1399 college students with symptom checklist 90 (SCL-90) and carried out correlation analysis and stepwise multiple regression analysis. The analysis found that the higher education level has a certain predictive value for the mental health status of college students, and there are significant differences between different groups, usually manifested in the trend that the higher the level of higher education, the better the mental health of college students. Ruan [1] believes that higher education managers should fully recognize the value of emotional management and make good use of the positive efficacy of emotional management, which can promote the mental health management of college students to a new level. Gong [4] conducted a questionnaire survey on 901 college students in Jinan. The analysis found that the severity of mental health problems of college students in all grades was significantly different. It was suggested that we should not only rely on the direct role of higher education itself, but also give full play to the subjective initiative of college students to improve their psychological conditions.

It can be seen from the above that most literature analyze the correlation between higher education level and college students' public mental health using traditional methods, such as questionnaire, mathematical statistics, and literature. But now, with the rapid development of artificial intelligence technology, artificial intelligence has been applied to the field of mental health. For example, the use of text, voice, facial expressions, and other multiple psychological data fusion, combined with a deep learning algorithm to evaluate the mental health problems of college students [5]. Use cutting-edge sensing technology, image and language recognition, big data analysis, etc. to quickly find students' psychological needs and help them actively respond [6]. However, there are still some deficiencies. Yang et al. [5] believe that due to different application scenarios, different data set sizes, parameter settings, and standard quality, and other factors lead to different results. As a result, the accuracy of prediction results of artificial intelligence technology varies greatly. This is because the subject of psychological data is human, which is more subjective than the data under natural science. Therefore, how to build a high-quality and reusable psychological database is a major challenge in this field. Guo and Hou [6] believe that AI psychological services still need to further improve the accuracy and comprehensiveness of physical and mental testing, obtain a wide range of validity verification data support, and design a more convenient application environment for areas and schools with relatively weak psychological service resources. Deng [7] combined the process of self-examination, self-diagnosis, and self-treatment of psychological problems, and launched an artificial intelligence diagnosis and treatment system for the whole closed loop. The system relies on computer software, a personal computer terminal and server to realize the penetration and connection of patients' remote self-test, teachers and students' remote communication, condition rehabilitation forum, and other functions. It mainly includes a health evaluation module, self-diagnosis and treatment module, treatment counseling module, tracking treatment module, and re-examination evaluation module. Li and Zhao [8] summarized the research status of the combination of artificial intelligence and psychological counseling technology. Nowadays, artificial intelligence psychological counseling can achieve professional nursing and companion support, psychological counseling, and real-time emotional dialogue feedback. At the same time, they detailed the theoretical content of the combination of the two, clarified the entry point of the application of artificial intelligence technology in psychological counseling, and summarized the methods and ways of its use and the evaluation of counseling effect, and the ethical issues of human–machine relationship. Wang et al. [9] believe that AI psychological counseling can complete the information collection, problem evaluation, and other work in the initial stage of psychological counseling. Moreover, AI psychological counseling can quickly integrate information from an unlimited number of medical information, generate mental health big data, and reveal the relevant disease behavior and pattern development trend through algorithm analysis.

The above research either uses traditional methods to analyze the correlation between the level of higher education and the public mental health of college students or applies AI to the field of psychology. This study attempts to apply AI technology to the analysis of the correlation between the two. First, the artificial intelligence technology is introduced, and the appropriate method is selected to analyze the correlation. Second, the current situation of college students' mental health is analyzed, and the influencing factors are selected to establish the correlation model based on BP neural network. Finally, the conclusion and discussion of this study are given.

## 2. Introduction to Artificial Intelligence Methods

The core technology of artificial intelligence is to analyze data by constructing a machine learning algorithm model. Machine learning is the study of computer simulation or realization of human learning behavior, reorganization of existing knowledge structure, and continuous improvement. Generally, machine learning algorithms can be divided into two categories: shallow learning and deep learning. Shallow learning algorithms such as decision tree, artificial neural network, and SVM have been widely used in the field of psychology, but the evaluation effect of their models is related to experience, so it is difficult to obtain good evaluation results. Deep learning is the machine learning algorithm with the best performance at present, which mainly includes a convolutional neural network, cyclic neural network, deep belief network, and other artificial intelligence methods. Deep learning comes from the artificial neural network. By establishing a deep neural network based on BP, it simulates the mechanism of the human brain to analyze and process all kinds of data [10, 11]. The deep learning represented by a deep neural network contains more hidden layers and can extract higher-level abstract features, which is the evolution and improvement of an artificial neural network. Zhou et al. [12] revealed the impact of noncoding mutation on autism spectrum disorder by building a model based on deep learning.

### 2.1. BP Neural Network

**Figure 1:**
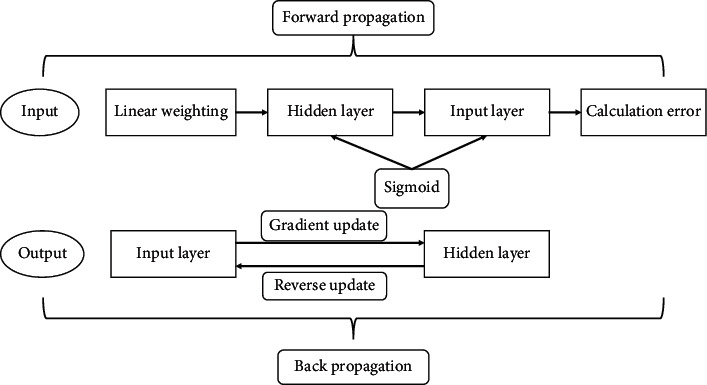
Forward and backward propagation mode of BP neural network.

BP neural network was proposed by Romero et al. [13] in 1986. BP neural network is built by multiple neurons. Its basic structure includes input layer, output layer, and hidden layer. It mainly realizes the learning mode through the hidden layer. After each learning, the information and results are stored in the network. BP neural network can obtain and adjust the model through two learning methods of signal forward propagation and error BP to achieve the optimal prediction effect. It is the neural network with the best prediction effect, the widest application, and the most mature development at present, as shown in Figure 1. BP neural network has no special requirements for data types and can solve nonlinear, nonnormal, discrete, and other multi-type data and variables. It is a big data mining method with high applicability. Compared with traditional early warning methods, BP neural network does not need any premise assumptions. The hidden layer of the network can be compared through many experiments to find the optimal number of neurons and hidden layers [14].

BP neural network shows method advantages in information storage and intelligent learning. BP neural network has multiple neurons, which can store a large amount of information and each learning result. It can continuously adjust parameters in the process of self-learning and has strong training ability and memory ability. Compared with other machine learning methods, BP neural network is more suitable for predicting a class of unknown problems. BP neural network can be operated in a dark box and has strong tolerance and fault tolerance, intelligent learning, and approaching any nonlinear problem; BP neural network is suitable for processing data with nonlinear, scattered, poor accuracy, and complex relationship characteristics. It has fast learning and processing ability for big data and meets the data type and analysis requirements of this study. BP neural network is a hot spot of nonlinear data mining technology. It processes data in a dark box in the whole process. It is suitable for data with fuzzy types, complex, and a large number of characteristics [15, 16]. The information trained by the network is stored with each neuron in the form of multiple groups of weights and thresholds to form network knowledge to evaluate or predict the outcome caused by relevant factors.

### 2.2. Decision Tree

The decision tree algorithm determines the classification of the samples in the data set by allocating the data samples to a leaf node and uses the tree model to recurse the sample space continuously. The tree model is composed of decision nodes, branches, and leaf nodes [17]. Each decision node in the tree represents a sample space. The connection between each decision node and leaf node is a path rule, and each leaf node represents a judgment category, which is mainly used to solve classification problems. The essence of the decision tree structure is to statistically classify the target data records according to the tree structure. Each leaf node of the decision tree represents a specific set of data records under certain conditions, and branches of the tree structure are constructed according to the values of data record segments [17]. The construction of a decision tree is composed of multiple nodes and directed edges. Generally, nodes can be divided into two types: internal nodes and external nodes. Internal nodes are composed of a single data attribute or a single feature, and their leaf nodes can also be considered as a certain class. Decision tree classification usually starts with the root node. In fact, it is to test the single characteristics of a certain instance and divide the specific instance into specific sub-nodes according to the test results. Thus, the specific instances can be effectively classified, and the information of different categories can be divided into the classes of leaf nodes. Setting *D* indicates the preset training data set:

(1) $$\begin{array}{c} D=\left\{\left(x_{1},y_{1}\right),\left(x_{2},y_{2}\right),\ldots \left(x_{n},y_{n}\right)\right\}\text{,} \end{array}$$

where *x* is used to describe the input instance, *n* is used to describe the number of instance features, *y* is the actual category mark, and *N* is the training sample size. The goal is to establish a decision tree model for a given training data set so that the model can be classified correctly. Generally, the circle describes the root node of the decision tree and the leaf node of the square second speed decision tree.

**Figure 2:**
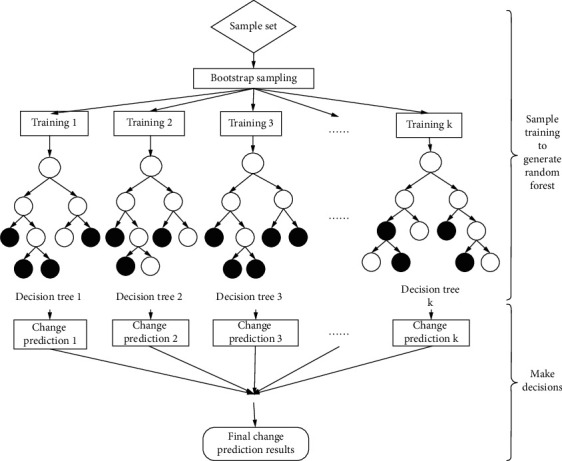
Schematic diagram of decision tree structure.

The decision tree described above can be regarded as a set of if-then rules. The root node of the decision tree connects each path of the leaf node step by step to construct a rule. The internal node characteristics of all paths have corresponding rule constraints, and the category of nodes in the decision tree corresponds to the conclusion of the rule [18]. Each path of the decision tree has corresponding properties: that is, mutually exclusive but complete, as shown in Figure 2.

According to the information theory described in the relevant literature, the smaller the expected information, the greater the corresponding information gain, and the higher the accuracy of the corresponding decision tree model. The decision tree algorithm [19] is: the attribute selection of the information gain measurement result is the final basis, and it is divided according to the attribute with the largest information gain after division. The specific practical concepts are given below.

Set *D* to represent the division of the training sample set by different categories, and the corresponding entropy of *D* can be described as:

(2) $$\begin{array}{c} H\left(D\right)=-\overset{n}{\underset{i=1}{\sum }}p_{i}\log_{2}p_{i}\text{,} \end{array}$$

where *p*_*i*_ is the probability that the first category appears in the whole training sample set, and *H* (*D*) is the information entropy. Set the training sample set *D* to be divided according to attribute *A*, and the expected information of using *A* to classify *D* can be described as:

(3) $$\begin{array}{c} H\left(D\right)=-\overset{n}{\underset{i=1}{\sum }}\frac{\left|D_{k}\right|}{D}\log_{2}\frac{\left|D_{k}\right|}{D}\text{,}\\ g\left(\left.D\right|A\right)=H\left(D\right)-H\left(\left.D\right|A\right)\text{.} \end{array}$$

The essence of the decision tree algorithm is to calculate the gain rate of each attribute in the training sample set at each split, and then select the attribute with the largest gain rate to split. In the split, set the cutoff condition to stop splitting when the number of data records of the current decision tree node is less than a given threshold.

### 2.3. Support Vector Machine

SVM is the most effective deep learning method to solve small sample and nonlinear regression, which was first proposed by Corinna and Vladimir [20] in 1995. SVM represents the sample data as points in space and uses kernel function transformation to transform the low-dimensional sample space into multi-dimensional sample space. In the high-dimensional space, the linear classification surface with the largest spacing space is taken as the decision boundary. The larger the distance between various sample points from the decision boundary, the smaller the classification error, and the best function with the largest deviation from the measured value of the sample is searched. Generally, *f* (*x*) can be expressed as:

(4) $$\begin{array}{c} f\left(x\right)=\omega \varphi \left(x\right)+b\text{,} \end{array}$$

where *ω* is the adjustable weight vector, *B* is the deviation vector, and then define the insensitive loss function:

(5) $$\begin{array}{c} L_{e}=\left\{\begin{array}{cc} 0\text{,} & y-\omega \varphi \left(x\right)-b\leq \varepsilon \text{,}\\ \left|y-\omega \varphi \left(x\right)-b\right|-\varepsilon \text{,} & other\text{,} \end{array}\right. \end{array}$$

where *ε* is the insensitivity coefficient, which is used to control the longitude of the fitting function, and *y* is the true value vector. By introducing nonzero relaxation variables, the regression problem can be transformed into a linear constrained convex quadratic optimization problem [13]. By introducing Lagrange multipliers, the linear fitting function can be obtained:

(6) $$\begin{array}{c} f\left(x\right)=\overset{n}{\underset{i=1}{\sum }}\left(a_{i}^{*}-a_{i}\right)\left(x_{i},x\right)+b\cdot \end{array}$$

For nonlinear problems, the kernel function *K* (*x*_*i*_, *x*) can be introduced to express the nonlinear situation as:

(7) $$\begin{array}{c} f\left(x\right)=\overset{n}{\underset{i=1}{\sum }}\left(a_{i}^{*}-a_{i}\right)K\left(x_{i},x\right)+b\cdot \end{array}$$

It can be found that the advantage of SVM is that it can classify and predict linear or nonlinear data. Its central idea is to find a plane in different dimensional data, and the two ends of the plane just divide the data into different categories. A kernel function is the core of SVM, which can map the low dimensional nonseparable data to the high dimensional space for classification, and construct the function that conforms to the characteristics between vectors. SVM is more suitable for the prediction of binary variable problems [21]. By finding the boundary of the data in the feature space, it can divide the plane or hyperplane in the low-dimensional or high-dimensional space to realize the classifier setting. And SVM has a strong anti-interference ability, which can be used in the noise learning part, and SVM can ensure the high generalization of the network and reduce the overfitting phenomenon of the neural network; SVM has a strong learning ability in the face of complex, nonlinear and high-dimensional data, and can use kernel functions to project irregular data into high-dimensional space. SVM can use classifier settings to learn data features.

### 2.4. Convolutional Neural Network

The convolutional neural network is an algorithmic mathematical model that imitates the structure and function of the human brain vision system [22]. It locally senses the outside world and extracts the local features of input data, and then uses the stacking of convolution layers to gradually expand the receptive field and complete feature extraction. Using the convolution calculation method, a convolution neural network can automatically extract features from the data set for image segmentation, such as color, gray, texture, and more abstract semantic features [23]. The core of learning these features is the continuous conversion of input data, and the output of a given pixel will be affected by a larger spatial neighborhood. Therefore, a convolutional neural network has achieved great success in speech recognition, image recognition, image segmentation, natural language processing, and other fields.

The convolutional neural network is actually a hierarchical model. The original data are gradually extracted from low-level features to high-level semantic features through the convolution layer, pooling layer, activation layer, full connection layer, and SoftMax layer of the network layer by layer. This study will use the method of deep learning to analyze the correlation between the level of higher education and college students' public mental health and compare it with the results of other shallow learning.

## 3. Artificial Intelligence Correlation Analysis Model

**Figure 3:**
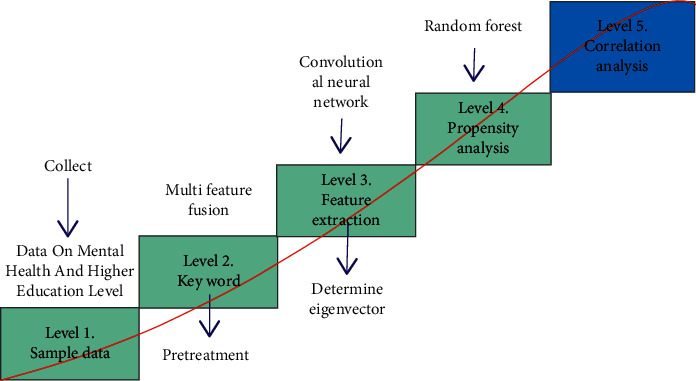
Structural framework of AI correlation analysis model.

In order to analyze the degree of interaction between higher education level and college students' public mental health and improve college students' understanding of their own mental health, this study uses the artificial intelligence method to analyze the correlation between them. The degree of correlation is divided into five dimensions: irrelevant, slightly related, medium, relatively related, and closely related. The AI analysis method of this correlation is mainly to build an AI correlation analysis model. The specific framework of the model is shown in Figure 3. The model is based on the collection of users' higher education information, focuses on the monitoring of mental health, relies on intelligent devices as the operation platform and data source, and deeply analyzes users' behavior information through users' heartbeat rate and movement data. At the same time, the status and comments of users' social accounts are extracted, and their emotional status and higher education level are analyzed. Through the information mining of multimodal data, we can detect the changes in users' psychological states in all aspects, so as to obtain the degree of correlation between the two combined with the information on higher education level [24].

In order to establish a comprehensive and intelligent correlation model between the level of higher education and college students' public mental health, this study constructs the original data set by collecting higher education information, college students' heart rate, social text, and other information, and extracts features respectively. Then, multi-feature fusion is used to further reduce the dimension of the feature vector. Finally, the fused feature vector is used as the training sample data to optimize the model parameters. In this study, the method of artificial intelligence is used to determine the feature vectors of physiological information and text information. For the classification and judgment of correlation, the random forest algorithm is used [25]. When the recognition accuracy of the model meets the threshold, it means that the model parameters have been trained and can be used to verify the test sample data. The random forest algorithm guarantees the diversity of the model by multiple random sampling to avoid oversaturation. At the same time, the sampling information is input into multiple weak classifiers to improve the complexity of the model. Voting on the classification results of each weak classifier ensures that the whole model has better classification accuracy and generalization ability.

### 3.1. Physiological Information and Mental Health

In recent years, more and more scholars began to pay attention to the relationship between heart rate and mental health, trying to use the change in heart rate to quantify mental health, so as to establish a quantitative description of college students' public mental health based on the change of heart rate. The change in heart rate is usually described by heart rate variability. Heart rate variability is defined as the change in the length of the time interval between successive heartbeats. Usually, the heartbeat of normal people does not maintain the same time interval, and when people are calm, exercise, and anxious, the heartbeat speed is also different. Therefore, by monitoring the heart rate of college students in different scenes, we can realize the quantitative detection of mental health.

The heart rate data is collected by the sensor of an intelligent wearable device, which is usually a series of time-varying and unsteady time-series waveform data. Therefore, it is necessary to calculate the time-frequency domain eigenvalue first, and then calculate the statistical characteristics of the data. Because people have different beating conditions in different states and different environments, integrating environmental information, sign state information, and behavior information into the mental health analysis model based on heart rate information will improve the recognition accuracy of mental diseases. The environmental information mainly includes the altitude and temperature of the tester's location; sign information includes body temperature and body surface humidity. The behavior information includes the number of walks, the change rate of the number of walks, and so on.

**Figure 4:**
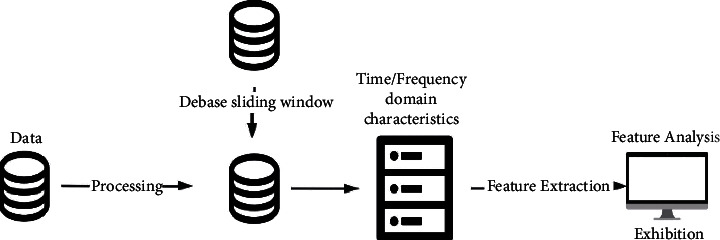
Schematic diagram of feature extraction and analysis of physiological information.

The above information is timing waveform data, and the feature extraction and analysis process are shown in Figure 4. First, the heart rate, environment, behavior, and somatosensory information are collected through the sensors of intelligent devices, and the discrete processing of different frequencies is carried out in order to reduce the amount of data and improve operation efficiency. Then, the white noise and meaningless data in the data are removed by debase and sliding window operations. Finally, feature extraction is carried out in the time domain and frequency domain respectively, and mathematical statistical calculation of each feature is carried out [26].

Considering the interference of noise signals caused by accelerometers and gyroscopes in intelligent wearable devices, it is necessary to preprocess the raw data collected by the sensor by using sliding windows and debase operation methods. In principle, the size of the sliding window should be set to twice the sampling frequency of the sensor, but in order to ensure the calculation of the fast Fourier function, the size of the actual window is defined as:

(8) $$\begin{array}{c} l=2^{ceil\left(\log_{2}\;2f\right)}\cdot \end{array}$$

Considering that the tester is not in motion all the time, and the static data account for a large proportion of the collected data, in order to reduce the amount of calculation of static data feature extraction, the study uses the debase operation to preprocess the static data. The specific operations are as follows: first, make the tester in a completely static environment, maintain a relaxed state, collect various data, and extract various features as benchmark values. When in other environments and user states, data samples can be used to subtract the benchmark value, so as to effectively reduce the impact of the environment and state and improve the calculation efficiency.

The time-domain characteristics mainly include mean value, standard deviation, maximum index, median, etc. Frequency domain features include DC component, signal amplitude area, and amplitude statistical features. The amplitude area of the signal can be described by the following formula:

(9) $$\begin{array}{c} SMA=\left(\frac{1}{t}\right)\overset{t}{\underset{0}{\int }}\left|x\left(t\right)\right|dt\cdot \end{array}$$

This index is defined as the sum of the area enclosed by discrete data and the time axis, which is used to distinguish static data from dynamic data.

In the data acquisition of motion state, the extraction of features should not only ensure accuracy, but also avoid excessive calculation. Therefore, (10) and (11) are used to calculate the eigenvalues in the time domain and frequency domain, where *i* represent the value of the *i*th accelerometer and gyroscope, AI represents the resultant acceleration, and *ω*_*i*_ represents the resultant angular velocity.

(10) $$\begin{array}{c} a_{i}=\sqrt{{\left(a_{i}^{x}\right)}^{2}+{\left(a_{i}^{y}\right)}^{2}+{\left(a_{i}^{z}\right)}^{2}}\cdot \end{array}$$

(11) $$\begin{array}{c} a_{i}=\sqrt{{\left(\omega_{i}^{x}\right)}^{2}+{\left(\omega_{i}^{y}\right)}^{2}+{\left(\omega_{i}^{z}\right)}^{2}}\cdot \end{array}$$

### 3.2. Text Information and Higher Education Level

**Figure 5:**
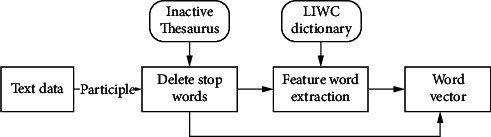
Extraction of feature vectors that integrate the text information of LIWC dictionary.

Physiological indicators such as heart rate are the performance of human physical state, while what people say and write is the reaction of their inner state and the level of higher education they have received. When college students have a higher level of education, their thoughts are more active than ordinary people. At the same time, this method can also be used to determine whether college students have mental health problems, because at this time, compared with ordinary people, their emotions have a certain degree of negativity [27, 28]. Therefore, the text information on the social platform is used to analyze the correlation between education level and mental health. The block diagram of the model is shown in Figure 5.

First, text features are extracted. Word bag model is used to decompose text and extract feature vectors. The key to word bag model lies in the construction of the dictionary and the weight calculation of each characteristic word [29]. In this study, the LIWC dictionary is used as the basic dictionary, which contains a large number of psychological process words, social process words, and language process words. By segmenting the text information of the user on the social forum, removing the stop words, and then comparing it with the LIWC dictionary, it is concluded that each word in the text information distinguishes the user's level of higher education and mental health.

Due to the different functions of different categories of vocabulary, the study mainly focuses on personal pronouns, negative words, cognitive process vocabulary, etc., and calculates the weight of these categories of vocabulary [30]. The specific process is as follows:

(1) First, count the number of occurrences of each part of speech in the relevant topics in the LIWC dictionary.
(2) For the above word frequency, calculate the standard deviation *a*_*l,i*_ and normalize the maximum value. The larger the value of the standard deviation, the more conducive it is to distinguish the emotional tendency and mental health embodied in the text information.
(3) Determine the weight of each word. The weight of the word in identifying the emotional tendency of the text is adjusted by judging which classification of the word belongs to the LIWC dictionary. The specific formula is shown in the formula
  (12) $$\begin{array}{c} b=\frac{\exp\left(a_{l,i}\right)}{\underset{l}{\sum }\exp\left(a_{l,i}\right)}+1\cdot \end{array}$$

**Figure 6:**
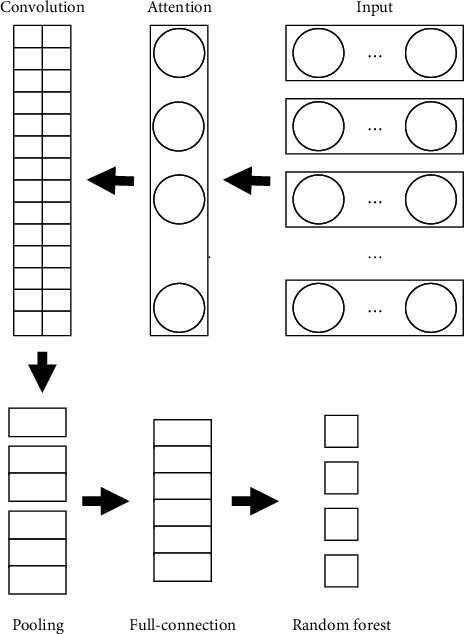
Structural diagram of text information emotional tendency recognition model based on random forest and convolutional neural network.

For the extraction of text word vector and text information tendency recognition, the text information tendency recognition model based on a random forest and convolution neural network is adopted, and the specific structure is shown in Figure 6. First, the feature vector generated above is input to the input layer in the form of a word vector matrix. In the attention layer, the standard deviation of different parts of speech can be calculated. The larger the standard deviation, the more significant the part of speech plays in identifying the level of higher education and mental health. In the convolution layer, different sizes of sliding windows are used to select local word vectors in the text, and then a new matrix is obtained by splicing [31, 32]. The pooling layer selects the most effective eigenvalue to distinguish the tendency by selecting the appropriate pooling function. The characteristic matrix in the full connection layer that has completed the above processing is transmitted to the random forest for classification. It is worth noting that the average generalization error PE of a decision tree in a random forest is related to the regression function, and the specific formula is shown in the formula:

(13) $$\begin{array}{c} PE\left(tree\right)=E_{\theta }E_{X,Y}{\left(Y-h\left(x,\theta \right)\right)}^{2}\cdot \end{array}$$

When using training samples for model training, the cross-entropy loss function is minimized by BP, and the weight coefficient of each neuron is regularized to avoid overfitting.

## 4. Experimental Test and Data Analysis

**Figure 7:**
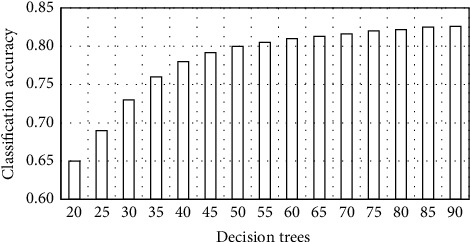
Influence of different number of decision trees on classification accuracy.

In order to verify the correctness of the method proposed in the study, the real data sets of multiple undergraduate, graduate, and doctoral students are used as the research objects, and their grades and gender are shown in Table 1. In order to ensure that the model training is sufficient and the results are effective, 85% of the sample data are randomly selected as the model training data. The rest of the data is used as detection data. The performance of the scheme described in this study is tested by setting a control group. The control group used BP neural network and SVM classifiers respectively. Because the number of decision trees in the random forest algorithm affects the classification accuracy, the number of decision trees is selected first. The test results are shown in Figure 7. It can be seen from the figure that with the increase in the number of decision trees, the classification accuracy gradually increases and tends to a fixed value.

**Figure 8:**
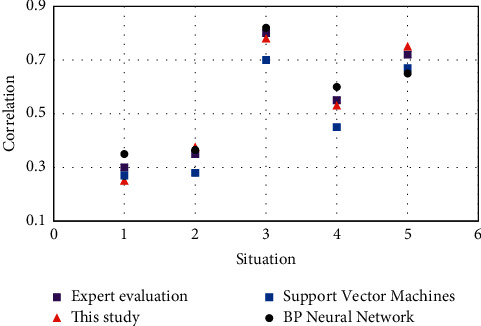
Comparison of recognition results between the experimental group and the control group.

Figure 8 takes the data of a research object as an example, and tests the degree of mental health in four ways: expert evaluation, random forest, SVM, and BP neural network. The horizontal axis “1,2,3,4,5” in the figure represents five kinds of correlation degrees: irrelevant, slightly related, medium, relatively related, and closely related. The closer to an expert evaluation in each dimension, the higher the recognition accuracy of the algorithm. It can be clearly seen from the figure that the recognition result of the random forest algorithm as a classifier is closer to expert evaluation. Based on all sample data, the statistical results are shown in Table 2. The accuracy of the recognition results of the scheme described in this study is 87.5% when training samples. The recognition accuracy of test samples is 86.3%. Both values are higher than those of the SVM and BP neural network algorithm.

## 5. Discussion and Conclusion

The level of higher education and the public mental health of college students are two important factors that affect the growth and development of college students. It is very important to explore the correlation between them. This can not only help college students further strengthen their studies, but also pay attention to the healthy and upward mind of college students. With the development of machine learning, it gives us a new way to solve this problem. This study introduces shallow learning and deep learning in artificial intelligence methods, in which deep learning mainly introduces convolution neural network, shallow learning mainly introduces decision tree, SVM and BP neural network, and determines to use convolution neural network and random forest algorithm in deep learning to analyze the correlation between higher education level and college students' public mental health. Using physiological information such as heart rate and social text information can quantify mental health and higher education level respectively, so as to provide a multi-level correlation analysis model. The application of a convolutional neural network makes the feature extraction of sample data faster and more accurate; At the same time, the random forest algorithm is used as the classifier. We selected nearly 2000 college students for analysis. After testing and data analysis, the established model has an accuracy of 87.5% in the determination and analysis of correlation. Compared with SVM (80.5%) and BP neural network algorithm (82.3%), it has a higher recognition accuracy.

## Figures and Tables

**Table 1:** Statistics of grade and gender of subjects.

Project	Undergraduate	Postgraduate	Doctoral student
Male	650	210	65
Female	580	220	75

**Table 2:** Comparison of correlation recognition results between experimental group and control group.

Project	Accuracy of training samples/%	Test sample accuracy/%
This study	87.5	86.3
BP neural network	82.3	83.5
SVM	80.5	79.8

## Data Availability

The experimental data used to support the findings of this study can be obtained from the corresponding author upon request.
